# 'A hidden disorder until the pieces fall into place' - a qualitative study of vaginal prolapse

**DOI:** 10.1186/1472-6874-10-18

**Published:** 2010-05-24

**Authors:** Mojgan Pakbaz, Margareta Persson, Mats Löfgren, Ingrid Mogren

**Affiliations:** 1 Department of Clinical Science, Obstetrics and Gynecology, Umeå University, 901 85, Umeå, Sweden; 2 Department of Nursing, Umeå University, Umeå, Sweden

## Abstract

**Background:**

Vaginal prolapse affects quality of life negatively and is associated with urinary, bowel, and sexual symptoms. Few qualitative studies have explored women's experiences of vaginal prolapse. The objective of the study was to elucidate the experiences of living with prolapse and its impact on daily life, prior to surgical intervention.

**Methods:**

In-depth interviews were conducted with 14 women with vaginal prolapse, prior to surgical treatment. Recruitment of the informants was according to 'purposive sampling'. An interview guide was developed, including open-ended questions addressing different themes, which was processed and revised during the data collection and constituted part of a study-emergent design. Data were collected until 'saturation' was achieved, that is, when no significant new information was obtained by conducting further interviews. Interviews were audiotaped, transcribed verbatim, and analyzed according to manifest and latent content analysis.

**Results:**

The theme defining the process of living with prolapse and women's experiences was labelled 'process of comprehension and action'. The findings constitute two categories: obstacles and facilitators to seeking health care. The category *obstacles *comprises six subcategories that define the factors restraining women from seeking health care: absence of information, blaming oneself, feeling ignored by the doctor, having a covert condition, adapting to successive impairment, and trivializing the symptoms and de-prioritizing own health. The category *facilitators *include five subcategories that define the factors promoting the seeking of health care: confirmation and support by others, difficulty in accepting an ageing body, feeling sexually unattractive, having an unnatural body, and reaching the point of action.

**Conclusion:**

The main theme identified was the 'process of comprehension and action'. This process consisted of factors functioning as either obstacles or facilitators to seeking health care. The main obstacles described by the participants were lack of information and confirmation. The main facilitators constituted feeling sexually unattractive and impaired physical ability due to prolapse. Information on prolapse should be easily accessible, to improve the possibility for women to gain knowledge about the condition and overcome obstacles to seeking health care. Health care professionals have a significant role in facilitating the process by confirming and informing women about available treatment.

## Background

Pelvic organ prolapse (POP) is associated with various symptoms such as urinary and fecal incontinence [1,2]. One study shows that POP has a significant negative impact on sexual function [3], while another reports that sexual activity and satisfaction are independent of pelvic floor disorders [4]. The effects of POP on quality of life have been extensively documented [5]. Although several quantitative studies have investigated these issues, qualitative research may be a complementary tool for capturing the perceptions and experiences of women with POP [6]. Further, understanding women's concerns and expectations are important factors for providing good health care services. The importance of investigating individual behaviour in relation to POP has been explored in some studies in an adjoining research area [7-9]. For women with recurrent symptoms in urogynaecology, barriers to seeking treatment have been elucidated and the identified factors include beliefs about ageing, tendency to minimize the importance of symptoms, and reluctance to use health service resources [9].

Few qualitative studies exploring women's experiences of vaginal prolapse have been performed. Learning about women's experiences of living with POP is important to increase understanding of the condition among health care professionals counselling this group of women, as well as to enable the subsequent postoperative evaluation.

The aims of the study were to elucidate the experiences of living with vaginal prolapse and its impact on daily life, prior a surgical intervention.

## Methods

### Participants

Fourteen women with symptomatic POP were recruited from the waiting list for prolapse reconstructive surgery of a hospital in northern Sweden. Symptomatic POP was defined as present of a vaginal bulge and/or other symptoms from the bowel or the bladder. Prolapse was assessed using the POP-Q classification system [10], and prolapse of stage II or more was identified at gynaecological examination. The inclusion criterion for the study was symptomatic vaginal prolapse for which corrective surgery was planned in the near future. Exclusion criterion was recurrent vaginal prolapse with previous surgical intervention. Recruitment of the informants with planned surgery was according to 'purposive sampling', i.e., the greatest variation of characteristics such as age, occupation, parity, BMI, and marital status in order to capture wide narratives from the informants (Table 1). The median age of the women was 56.5 years (range 42-79 years). Parity ranged from one to five and no informants included in the study reported a history of assisted delivery. Duration of symptoms of vaginal prolapse reported by the women varied from 2 to 10 years. Sixteen consecutive women were asked to participate; 14 agreed to take part in the study. Only one informant had a patient-surgeon relationship with the first author. The informant having a patient-surgeon relationship was the youngest woman. Considering the 'purposive sampling' approach, the research group decided to recruit informants with as wide a range of age as possible (both ends of the age span). Therefore, an exception was made to include this informant, although the first author had medically counselled this patient at a single occasion previously, and the patient-surgeon relationship was discontinued after the interview.

**Table 1:** Background characteristics of the participants.

No.	Age	Parity	BMI*	Occupation	Marital status
1	55	2	24.6	Teacher at university	Married
2	69	3	21.3	Senior citizen	Married
3	79	2	22.4	Senior citizen	Married
4	47	2	22.3	Office employee	Single
5	58	5	21.8	Health professional	Married
6	60	4	26.4	Teacher at music school	Married
7	51	4	25.1	Teacher	Cohabitant
8	56	3	24.2	Self-employed	Married
9	79	3	26.5	Senior citizen	Widow
10	59	2	31.0	Art teacher	Married
11	57	2	25.8	Manager	Married
12	56	4	29.0	Cook	Single
13	42	4	21.4	Office employee	Single
14	54	1	23.3	Nurse	Married

*Body mass index (BMI)

### Interviews

An interview guide was developed by the investigators, including open-ended questions addressing different topic areas (Table 2), based on the scientific literature of determinants for vaginal prolapse and the clinical experiences from this medical field available within the research group. The interview guide was thereafter processed in the research group in order to integrate as many appropriate aspects of the research question as possible. The interview guide was later revised during the data collection, according to an emergent study design. The research group consisted of an urogynaecologist (MoP), a midwife (MaP), a gynaecologist (ML), and an obstetrician (IM).

**Table 2:** Template for semi-structured interview.

Topic area	Open-ended questions
Debut of the symptoms	When did you notice the first symptoms of your vaginal prolapse?
Symptoms	Can you describe your symptoms of the vaginal prolapse?
Social life Working life Leisure time Sexual life	Can you explain how the vaginal prolapse affects your life?
Knowledge/information	Can you explain what the condition vaginal prolapse is and do you know the causes? What do you think about the availability of information regarding vaginal prolapse in society?
Communication	With whom do you communicate about your vaginal prolapse?

The potential informants were contacted by phone by the first author (MoP). Initially, they were orally informed about the purpose and method of conducting the study, and asked to take part. Subsequently, an information letter was delivered to the informants willing to participate, which also emphasized that participation in the study was voluntary, and that they could withdraw whenever they wanted during the course of the study without affecting their further treatment. Verbal informed consent was obtained from all informants before the interview was conducted. The privacy of women and the confidentiality of the collected data were assured. The informants were given free choice to decide the place for the interview. All but one interview took place in a separate room within the hospital. One interview was performed in the home of the informant. In-depth interviews lasting 40-50 minutes were conducted. The interviewer specifically stated to the informants at the beginning of each interview that the interview was a research event, not a medical consultation. The interviewer strived to create an atmosphere of interest and respect for the experiences conveyed by the informant. All communication skills were used to ensure an open discussion and to encourage the informants to fully describe their experiences. By using follow-up questions like 'Could you tell me more about it?' and 'What do you mean?' or 'Would you please give an example?' the experiences of the informants were further elaborated. At the end of each interview, the investigator made a verbal summary of the informant's statements to ensure that the history given by the informant had not been misunderstood. The first three interviews were conducted by two investigators; the second author (MaP) was responsible for the interviewing, while the first author (MoP) acquainted herself with the interview technique. The next three interviews were conducted by the first author, while the second author was the observer. The first author transcribed verbatim the first three tape-recorded interviews. Pauses and interruptions were also noted. The remaining interviews were transcribed by a secretary who used the same approach and procedure in the transcription process as the first author. After eight interviews with concurrent analyses, a revision of the interview guide was made and an additional four informants were recruited to the study, thus constituting an emergent design. At this stage when twelve interviews were performed, saturation of data related to the research question was obtained; however, two final informants were recruited to ensure that no new substantial experiences were expressed.

### Data analysis

The interviews were analysed using qualitative content analysis [11]. Both the manifest content, that is, what the text said [11,12], and the latent content, what the text described and expressed [11,12], were analysed. Qualitative content analysis focuses on the subject and the context illustrating the differences between, and similarities within, codes and categories [11]. Content analysis is a stepwise process. First, the interviews were thoroughly read several times to obtain a sense of the whole and identify the content area. Second, the text was divided into meaning units and condensed to make the text shorter, while retaining its core message. At the third step, the condensed meaning units were abstracted and labelled with codes. Fourth, the various codes were compared on the basis of similarities and differences and sorted into schemes labelled categories and subcategories. The final step of the analysis was to identify a theme.

In order to seek consensus between the researchers, the categories and subcategories were further discussed, which led to refinement of the scheme of categories, and the identification of a single theme, which was interpreted as the underlying or latent message of the abstracted categories. The study was approved by the Ethics Committee, University of Umeå, Sweden (Dnr 08-076).

## Results

The theme defining the process of women's experiences of living with prolapse and its influence on daily life was labelled the 'process of comprehension and action'. Two categories (*obstacles *and *facilitators*) and 11 subcategories emerged from the data (Table 3). Subcategories were categorized as either obstacles or facilitators, according to their main features during the process. Some of the subcategories also exerted the contrary effects, but to a lesser extent during the earlier phase of the process.

**Table 3:** Theme, categories, and subcategories describing the experiences of women with vaginal prolapse.

Theme	Category	Sub-category
Process of comprehension and action	Obstacles to seeking health care	Absence of information
		Blaming oneself
		Feeling ignored by the doctor
		Having a covert condition
		Adapting to successive impairment
		Trivializing of symptoms and de-prioritizing own health
	Facilitators to seeking health care	Confirmation and support by others
		Difficulty in accepting an ageing body
		Feeling sexually unattractive
		Having an unnatural body
		Reaching the point of action

### Obstacles to seeking health care

The category obstacles to seeking health care include six subcategories, with factors that exerted a negative influence on seeking health care.

#### Absence of information

Informants expressed that information on vaginal prolapse was not easily accessed. They had not observed any information about prolapse in brochures or weekly magazines by accident. They had to actively search for information on prolapse. Insufficient knowledge about the condition at an individual level, as well as within the societal arena, made the informants uninformed and uncertain. They couldn't address which condition they were actually suffering from.

There isn't any information really [on prolapse], not in the way that you just stumble across it somehow. If I haven't searched [for the information] myself, I don't think I just happen to read about it by accident (participant no. 1).

You have to search for information [about prolapse], because there isn't anything, really. There is more [information] about urinary incontinence and incontinence sanitary pads; otherwise, there is no information about prolapse (participant no. 5).

I think there is very little written about this problem [prolapse] if you read just about anything, like weekly magazines. You know, you can read a lot about genital problems in magazines, but this particular problem I haven't run across often. I didn't even know there was another condition called prolapse, besides when the uterus falls out. It was the only kind of prolapse I knew existed (participant no. 13).

#### Blaming oneself

Several informants considered themselves to be physically strong; they were not afraid of hard work and regarded themselves as physically independent of others. They reported that they exposed themselves to extensive physical work and felt joy and pride, due to their physical abilities. They were enterprising and active. They considered that their impediment with prolapse was going to be overcome. They reported that they seldom requested help and did not complain about small matters.

Some informants were not surprised at having a problem with prolapse, because of all heavy physical work they had performed previously in life. They assessed that they had themselves to blame and did not want to bother the health care services with their problems. Some informants felt guilty that they had ignored pelvic floor training after the childbearing period and wondered if they could have prevented the prolapse by actively engaging in pelvic floor muscle training. Others' opinions about causes of prolapse made informants feel guilty.

When something has to be done or moved [in the cottage], you don't bother to call around and ask for help. Instead you do everything yourself, like moving furniture, and do everything, like pull out the sink and turn the dishwasher upside down before winter so that it [dishwasher] will not freeze. All such stuff [heavy lifting] I do myself, because it is such nuisance to gather some men to help. So, it's easiest to do everything yourself (participant no. 10).

I am hard-working, to the extent that I do anything. That means that if I have decided that something has to be done, I will do it no matter how I feel [ignoring physical signals]. We have solar cells with huge batteries, so heavy that you can hardly lift them, but you lift them, anyway (participant no. 3).

I worked hard in the cowshed. So I think you have... Well... I worked hard and haven't taken any rests [talks about what may have caused the prolapse] (participant no. 2).

I have worked hard in the forest, so I have taken more than the body can take, really (participant no. 2).

#### Feeling ignored by the doctor

The informants reported having had symptoms of prolapse several years before they decided to seek health care for the problem. Some informants expressed that their symptoms had not been confirmed at previous consultations with doctors, which made them doubt their own perceptions of the condition. They expressed that the inconvenience they felt was not acknowledged and confirmed, leading to a further delay until they generated enough motivation and courage to visit the doctor again. Several of the informants experienced that they had not received information from their doctor on the procedure for the planned operation.

When he [the doctor] had seen it [in earlier consultation], he thought, 'This isn't a problem'. I was disappointed, so, 'It wasn't so bad'? I was a bit surprised, and I feel that I have problems. But I believed [what the doctor said], 'There is no problem' so I carried on. That's why I went so long [before seeing the doctor again] (participant no. 6).

I contacted you [the gynaecological outpatient ward] five years ago. It felt pressing... I felt heaviness, and it felt unnatural. The condition [prolapse] was not treated at all, so I took no contact until four years later, when the prolapse started to emerge out of the vagina (participant no. 9).

#### Having a covert condition

As the condition was covert, the participants had not previously considered that it could be prolapse. Despite experiencing problems, they were not aware of the deviating condition, until the vaginal mucosa was exposed. Further, the prolapse was invisible to others; no one else knew that the informants had a specific problem with prolapse, until they reported their condition to others. Some informants expressed feelings of shame to reveal their problems, and expressed that it was an intimate issue to discuss with others. The condition, itself, was reported as painless by most of the participants.

It [prolapse] is a rather covert condition, because you don't talk [about it]. You don't discuss it during a dinner party, 'Well, my uterus is prolapsing'; you just don't say that (participant no. 7).

It is sort of unpleasant to talk about it [prolapse]. Not nefarious, but close to. Somehow, it is more sensitive to talk about one's intimate parts (participant no. 9).

As this condition is internal and invisible--I mean when guys have a problem with their testicles, it is visible and obvious, hence less taboo, because then you see it. But you can't see this [prolapse]; that's why you don't approach people and ask about it (participant no. 13).

#### Adapting to successive impairment

The informants described different strategies for coping with symptoms of prolapse. Successively as the symptoms increased, the informants made conscious or unconscious adaptations for symptom relief. Some informants assisted digitally (with fingers via vagina) to empty the bowel of faeces, or remained sitting on the toilet for longer periods. Another coping mechanism was changing diet to overcome the problem with constipation. 'Holding back the lump' was an additional strategy used in order to urinate or defecate. Some informants chose to wear tight underwear to 'keep the lump in place'. Furthermore, the women felt restricted in their social lives and would not perform certain activities spontaneously. They expressed that the symptoms of prolapse increased with heavy lifting, and therefore, they requested relatives to help with, for example, grocery shopping and heavy activities. They changed to other kinds of physical activities that were less straining, such as cycling, instead of walking or jogging. If concurrent urinary urgency were a symptom, the women made a point of locating toilets whenever they were in a public area, in order to have quick access, if necessary. Some informants chose alternative positions during intercourse to make the sexual act more convenient.

I can't relieve myself without holding back the prolapse with my hand. It is very unpleasant. When I strain, it [the prolapse] is coming out, so I have to hold it back (participant no. 9).

You have to constantly think twice; don't do anything abruptly, and plan, especially now when I have become incontinent. You have to plan all the time, to be prepared for anything, which means not being able to do something spontaneously" (participant no. 14).

I have to lie down on my back [during intercourse], because it [the prolapse] is hanging out, otherwise it is not possible [to have intercourse] (participant no. 3).

#### Trivializing of symptoms and de-prioritizing own health

The informants tended to trivialize their symptoms of prolapse. Some women assessed that prolapse was a condition of relatively little importance, when compared to other medical conditions, or other problems they experienced in their social lives. One informant considered whether she actually had the time to undergo surgery, due to the long period of convalescence. Insufficient financial compensation during the sick-leave period after surgery, being a regular client at the gynaecological outpatient ward for other reasons previously, or being acquainted with some of the health care professionals were described as other counteracting factors. Prolapse was not considered by the women as a life-threatening condition, and furthermore, feelings of discomfort about gynaecological examinations were reasons some the informants waited to seek treatment. Informants who felt their sexuality unaffected by the prolapse tended to minimize the symptoms they experienced.

I have heard it is a long convalescence [after surgery], and then maybe you think automatically, 'Do I have the time for this?'(participant no. 11).

My problems [with prolapse] are small. That you have to admit, when you see all the misery in the world (participant no. 10).

### Facilitators to seeking health care

The category facilitators to seeking health care include five subcategories describing factors that promoted seeking the health care (Table 3).

#### Confirmation and support by others

Informants made an active choice whether to communicate their condition to others. They chose to discuss the matter with individuals they had confidence in or who they knew to have professional knowledge of the subject. Through conversation with others who were more knowledgeable about the condition, the informants increased their knowledge. Some informants communicated with others only after a gynaecologist had confirmed their condition and the surgical intervention was already planned.

Informants with current partners commonly discussed their condition with them. Some informants reported that their partners encouraged them to seek health care services because they had noticed the informants' difficulties in dealing with prolapse and their unwillingness to have intercourse. Some partners had pointed out the anatomical deviation, as they perceived it during intercourse.

My husband wants me to have surgery as soon as possible, so I will be okay. He says, 'You can't go on with this [prolapse]' (participant no. 3).

You don't go around far and wide and talk about it [prolapse], but you can do it in the right context (participant no. 6).

I have two cousins who are midwives, and when I explained my problems [with prolapse] to my cousin, and she had listened, she said 'Well, I think you have prolapse' (participant no. 4).

#### Difficulty in accepting an ageing body

Prolapse was interpreted by some informants as a sign of ageing; however, they did not feel old themselves. They had difficulties in accepting the body 'turning old' and dreaded not being sexually attractive. One informant expressed that she did everything possible to keep her body in shape, but did not succeed.

On the whole, I might have difficulties about this (getting old), and it is not just about this function inside of me [vaginal changes due to prolapse]. It is also complexional, getting skin changes, these things. I suppose it has to do with my marital status; I live alone and I still want to be attractive (participant no. 4).

Older women should be developing it [prolapse], I am young. I am 55 [years] soon (participant no. 1).

#### Feeling sexually unattractive

Prolapse affected some informants' self-esteem negatively and they expressed that they felt they were not sexually attractive to their partners. There was an uncertainty about how the intimate parts of the body were going to react during intercourse, compared to the period before prolapse developed. The experienced alteration in the intimate parts of the body sometimes made intercourse unpleasant for them. One informant expressed that she perceived herself as not sexually attractive for her partner. Some single women found it unthinkable to start a sexual relationship as long as they suffered from prolapse, and therefore they resisted. Feelings of sexual unattractiveness were expressed more frequently by the relatively young and single women among the informants.

I would have difficulties imagining having sex with any man now. It would be rather awkward or it wouldn't work as well as before (participant no. 12).

I don't feel like having sex, at all. It's a feeling of discomfort, because I know the way my body [vagina] looks [because of prolapse] (participant no. 1).

I don't feel [sexually] attractive. I have lost part of myself as a sexual being (participant no. 7).

#### Having an unnatural body

Informants had actively inspected their vulva region and interpreted the body change as 'a lump coming out'. Prolapse was described as an uncomfortable and unnatural condition, and the informants were continually reminded of their adverse condition, due to the prolapse. Furthermore, there were alterations in the function of the bladder and the bowels, and as a result of the inconvenience caused by these changes, the informants desired to regain their previous normal function of these organs.

For some informants, the symptoms of prolapse became more perceptible at menopause. Vaginal dryness became more distinct. Vaginal heaviness was further pronounced in a standing position and by the end of the day. Informants described different symptoms associated with the experiences of prolapse, such as difficulty in bowel evacuation, urinary urgency, feeling of urinary retention, persistent urinary tract infection, urinary incontinence, feeling of pain and friction, and waking up in the night with urgency to urinate. Urinary incontinence was experienced as shameful, and the most significant symptom that made them to seek health care services.

It's like the [vaginal] wall is inside out; it is like an oval egg. It is very uncomfortable, as if the labia are apart (participant no. 3).

If I look between my legs, it looks like as if the bowel is coming out [from the vagina]. Well, it is like a lump that is protruding, and if I push hard even the cervix is protruding. I can extract my IUD string with my fingers (participant no. 4).

When I need to go to the bathroom, it is just like I am giving birth through my anus; it feels like I'm passing a big melon (participant no. 4).

I never feel completely clean. You're afraid of smelling [urine], and it's not good to wash with soap too often. The pad has to be changed frequently (participant no. 3).

#### Reaching the point of action

According to several informants, there was a delay in seeking health care services. In the initial phase, the symptoms could be managed; however, as symptoms successively increased and the women perceived the restriction of activities in their daily life, they reached the point where they could not stand the situation. Sometimes a trigger event (for example, postmenopausal bleeding) was the factor that accelerated the process of seeking health care, while for some informants a trigger person (for example, a midwife) confirmed their discomfort with the prolapse and encouraged them to consult a gynaecologist. Some informants reported that they sought medical care because they did not want to wait until they got older and might have a deteriorated prerequisite for surgery.

Maybe two and a half years ago, I was visiting the midwife for a routine examination [pap smear] and she told me, 'Your prolapse is large and you should visit a doctor', but I didn't. Last autumn when I was raking [in the garden], I had vaginal bleeding [postmenopausal], therefore, I contacted you here, so I could have a gynaecological examination (participant no. 5).

## Discussion

The current study investigated the experiences of vaginal prolapse and its impact on daily life, prior to surgical intervention. The study identified the process from recognition of prolapse to action, which is, seeking health care. The two main categories, labelled *obstacles *and *facilitators*, included subcategories acting as barriers or promoters to actively seeking health care. Subcategories associated with a category had their main features as facilitator or obstacle during the process. Some of the subcategories also exerted the opposite effects, but to a lesser extent.

Prolapse influenced several aspects of the women's life. Although vaginal prolapse is relatively common [13], it was surprising that women in this study expressed substantial lack of knowledge about the condition. Deficiency of knowledge about prolapse and feelings of shame were important factors in the delay in seeking health care services. Therefore, the insufficient societal knowledge described by participants in this study needs to be addressed. Lack of knowledge about these issues has been shown in other studies [8,14] to play a key role in a patient's behaviour with respect to seeking health care. Women in general could benefit from more information about prolapse and the treatment options available. Providing information leaflets would probably make women more aware of prolapse and possibly facilitate the process of adequately addressing their symptoms by seeking health care.

In one study [9], barriers to seeking treatment for women with recurrent urogynaecological symptoms were investigated with Grounded theory, and in contrast to our study, women were already information about the condition and factors such as shame and embarrassment might have had less influence. However, consistent with the current study, women tended to minimize the importance of symptoms. Another study using phenomenological qualitative analysis to explore women's experiences of genital prolapse [8] reports that women expressed insufficient knowledge about their condition and their self-image also was affected. In a population-based study, factors like older age, hormone use, hysterectomy, and frequent urinary tract infection were found to be significant factors for seeking health care among women with pelvic floor disorders [15].

In the current study, different strategies and coping mechanisms resulted in adaptation to the condition, and we found that these coping mechanisms may have a negative impact on the action of seeking health care. Another possible explanation for the delay in seeking health care might be that the symptoms were not perceived as troublesome in the beginning, and by finding different coping strategies, the women adjusted to the condition, although it progressively worsened. The condition was reported as not painful for most of the participants, which could have been a contributing factor in the delay in seeking health care.

Furthermore, some women reported that their symptoms of prolapse were sometimes ignored by doctors, resulting in a further delay of the process of seeking health care. However, later on, a trigger event caused them to consult a doctor once more. From this study we may conclude that health care professionals may have a significant role in providing women with adequate information and confirmation of their symptoms.

Patient-centred goals have been advocated as a method of evaluating outcomes in urogynaecology [16-18]. In this study, the discomfort of the gynaecological examination was a barrier to seeking health care for some women, and hence, a reason for not consulting a gynaecologist when the first symptoms related to prolapse occurred. A mutual discussion of goals that may be achieved by treatment, thereby, setting realistic expectations, may enhance the patient-clinician communication and improve patient satisfaction. In a Cochrane meta-analysis [19] investigating the efficacy of decision aids for people facing health treatment decisions, the conclusion is that decision aids, such as pamphlets, increase patients' knowledge of treatment options and their involvement in the decision-making process.

Women with prolapse report decreased body image and quality of life [20]. In our study, significant factors facilitating informants' seeking health care were feelings of being sexually unattractive, impaired physical ability, and moreover, a sense of getting old, which was most prominent among the younger informants, and even more among the single women in our study. Further qualitative studies in this research area should investigate whether anatomical improvement by treatment may improve sexuality. Furthermore, the deficiency of knowledge and the patient-physician interaction regarding appropriate information before treatment needs to be further explored.

### Methodological considerations

Our aim was to achieve a deeper understanding of women's experience of vaginal prolapse. The approach in qualitative research focuses on individuals' experiences and may provide rich and detailed descriptions of previously unexplored phenomena [21]. As a method, qualitative content analysis offers a fairly straightforward method of analysing textual data and provides some specific norms and guidelines for data analysis [11]. The researcher may, for instance, choose to make the analysis more or less manifest or latent [11,12], the latter leading to a more interpretive approach. The described procedures during the analysis contribute to transparency of the analytical method and the analyst's thought processes. This enhances the scientific rigour of the research [11]. Interview data are constructed jointly by the interviewer and the interviewee. The main interviewer (MoP) was an experienced urogynaecologist. There is a risk for the pre-understanding of the researchers to influence the analysis and the interpretation of the findings. However, the pre-understanding most probably contributed positively to the quality of the data collection. The co-authors were a midwife (MaP), a gynaecologist (ML), and an obstetrician (IM). The second and last author had previous substantial experience of qualitative research. The analysis procedure has been discussed thoroughly within the research group in order to challenge potential pre-understanding. The different professional backgrounds may have improved the validation of findings. As all informants in the study were slated for surgical intervention of their vaginal prolapse, these results may be generalized to the experiences of other women in the same situation. However, other women with vaginal prolapse, identified or unidentified, but not in need of surgery, may have other experiences that expand the spectrum of experiences of vaginal prolapse.

### Trustworthiness

In qualitative research, the findings are evaluated in terms of trustworthiness, which comprises credibility, transferability, dependability, and confirmability [11]. An important aspect of credibility is to present the data and the process of analysis. During the categorization, the first author read and reread the text repeatedly in order to get immersed in the data. To further enhance credibility, all researchers in the team were involved in the process of analysis, through continual discussions of the findings. Trustworthiness was further enhanced in this study by independent categorization by colleagues, as proposed by Burnard [22]. No member checks (also known as respondent validation) were conducted, since the study results were synthesized, decontextualized, and abstracted from individual characteristics; hence, the informants might not recognize their contributions to the study [23].

Issues related to trustworthiness were addressed throughout the research process, for example, by leaving a decision trail [24], discussing theoretical, methodological, and analytical choices. Leaving a decision trail may be a way to enhance confirmability. To further visualize the interpretation of findings, quotations from the text were used to illustrate the experiences of the informants.

The interviewer was the instrument of the data collection. As the study progressed and the researcher's interview technique developed, the quality of data also improved. To establish dependability, an open dialogue between researchers was created and interpretation of the data was discussed. The researchers' judgments about the new data obtained during the study period and similarities and differences of the content were consistent over time. Referring to the issue of transferability, the experiences described by the informants in this study may be transferred to other women in similar settings awaiting surgery.

## Conclusion

The main theme identified was the 'process of comprehension and action'. This process consisted of factors functioning as either obstacles or facilitators to seeking health care. The main obstacles were the lack of information and confirmation. The main facilitators constituted feeling sexually unattractive and impaired physical ability due to prolapse. Information about prolapse should be easily accessible, to improve the possibility for women to gain knowledge about their condition and overcome obstacles to seeking health care. Health care professionals have a significant role to facilitate the process by confirming, informing, and counselling on available treatment options.

## Competing interests

The authors declare that they have no competing interests.

## Authors' contributions

MoP was involved in data collection, study design, data analysis and interpretation, and drafting and writing of the manuscript. MaP was involved in data collection, study design, data analysis and interpretation, and drafting of the manuscript. ML participated in the data analysis and interpretation. IM was involved in study design, data analysis and interpretation, and drafting of the manuscript. All authors read and approved the final manuscript.

## Pre-publication history

The pre-publication history for this paper can be accessed here:

http://www.biomedcentral.com/1472-6874/10/18/prepub
